# Lipoprotein(a)'s Role in Atherosclerosis and Aortic Stenosis: A Contemporary Literature Review

**DOI:** 10.7759/cureus.62984

**Published:** 2024-06-23

**Authors:** Mukosolu F Obi, Manjari Sharma, Shardil Ahmad, Safi Ur Rehman Daim, Ngozi T Kanu, Luis Diaz

**Affiliations:** 1 Internal Medicine, Wyckoff Heights Medical Center, Brooklyn, USA; 2 Internal Medicine, St. George's University School of Medicine, True Blue, GRD; 3 Internal Medicine, Mayo Hospital, Lahore, PAK

**Keywords:** cardiovascular disease (cvd), ldl cholesterol, oxidative stress, atherosclerosis, lipoprotein (a), aortic stenosis (as)

## Abstract

Lipoprotein(a), or Lp(a), is a distinctive lipoprotein particle linked to various cardiovascular diseases, notably atherosclerosis and aortic stenosis. Much like plasminogen, Lp(a) hinders normal fibrinolysis, leading to increased thrombosis and slower clearance of fibrin debris. It also causes inflammation, oxidative stress, and endothelial dysfunction, contributing to the formation of atherosclerotic lesions. Epidemiological studies have consistently shown that even slight increases in Lp(a) levels correlate with a heightened risk of cardiovascular events. Furthermore, Lp(a) plays a role in aortic stenosis by binding to leaflet valves, accumulating within them, and triggering calcium deposition and nodule formation. These calcium deposits gradually narrow the arteries, impeding blood flow. By raising inflammation and oxidative stress in the valve, Lp(a) accelerates tissue damage and calcium deposition. Traditional lipid-lowering therapies have limited efficacy in reducing Lp(a) levels. However, new treatments using RNA interference and antisense oligonucleotides to decrease Lp(a) production in the liver offer promising prospects for mitigating the risks and managing atherosclerosis and aortic stenosis associated with high Lp(a) levels. As Lp(a) screening becomes more common in healthcare, physicians will be better equipped to assess patients' risk levels and provide tailored treatments. This review aims to examine the role of Lp(a) in the development of aortic stenosis and atherosclerosis.

## Introduction and background

Lipoprotein(a), or Lp(a), has been identified as playing a significant role in cardiovascular disease, including atherosclerosis and aortic stenosis. This complex lipoprotein is structured with a low-density lipoprotein (LDL)-like particle in addition to an ingredient called apolipoprotein(a) (apo(a)), which is covalently attached to an apolipoprotein B100 on the LDL particle [1]. Interestingly, Lp(a) is structurally similar to plasminogen, a compound critical in blood clot breakdown [2]. This structural similarity is argued to play a pathogenic role in the Lp(a) interaction through its interference with the normal fibrinolytic process, leading to increased thrombosis and poor fibrin clot clearance. Furthermore, Lp(a) is known to induce inflammation, oxidative stress, and endothelial dysfunction, all of which are implicated in the development of atherosclerotic lesions [3]. Atherosclerosis, an inflammatory condition in which cholesterol plaques accumulate in the arteries, is a significant contributing factor in cardiovascular diseases (CVDs) such as coronary artery disease, cerebral vascular diseases, and peripheral artery diseases.

Lp(a) plays a crucial role in the development and progression of atherosclerosis through multiple mechanisms [4]. One key aspect is its ability to penetrate arteries and bind to extracellular matrix components like fibrin, glycosaminoglycan, and proteoglycan [5]. This phenomenon of binding drives the Lp(a) sequestration in the arterial intima, where it can undergo oxidative modification and consequently prompt the influx of such inflammatory cells as monocytes and macrophages into the lesion [4]. These inflammatory cells then pick up oxidizing Lp(a) particles bit by bit, which causes the formation of foam cells, the underlying pathological feature of early atherosclerotic lesions [2]. Lp(a) is well established as causing endothelial dysfunction, which is acknowledged as an early happening in atherosclerosis development [3]. Deficient endothelium can then be identified by resistance to vasodilation, abnormal lipoprotein passage through the endothelium, and elevated expression of adhesion molecules that stimulate cells to migrate to the arterial wall [1]. Furthermore, Lp(a) may contribute to the development of atherosclerosis by accelerating the oxidation of LDL, which causes the formation of oxidized LDL (ox-LDL) particles [6]. Ox-LDL is exceptionally atherogenic, which stimulates the production of proinflammatory cytokines, thereby worsening the inflammatory response within the artery's wall [2].

Besides the contributions of Lp(a) to the development of atherosclerosis, Lp(a) has been identified as a significant contributing factor to aortic stenosis, a condition in which the aortic valve opening narrows gradually. Development of aortic stenosis is accompanied by left ventricular hypertrophy, heart failure, and death if not treated promptly. How Lp(a) participates in developing aortic stenosis is not well understood, and more mechanisms still need to be proposed [7]. One model for the role of Lp(a) in aortic stenosis identifies Lp(a) as a protein that can bind and accumulate within the aortic valve leaflets, which causes calcification and formation of nodules [8]. This can be a gradual process whereby calcium deposits eventually build up, locking the valve's leaflets and obstructing blood flow [6].

## Review

The aims of this literature review are to critically assess and integrate the current findings on the link between high Lp(a) levels and the emergence of atherosclerosis and aortic valve disease. This review investigates how Lp(a) is likely involved in the pathologic processes leading to these cardiovascular diseases; such as its role in inflammation, oxidative stress, endothelium dysfunction, and calcium morphogenesis. We also examine how an augmented Lp(a) level affects cardiovascular risk and its impact on the development of risk stratification and personalized treatment strategies, analyze the current and novel therapeutic plans shown to reduce Lp(a) levels and evaluate their effectiveness in lowering the risk of CVD caused by high Lp(a).

Awareness of the link between high levels of Lp(a) and CVD is vital for several reasons [9]. Lp(a) is a highly atherogenic lipoprotein particle that has continually been recognized as an independent risk factor for conditions such as atherosclerotic cardiovascular disease (ASCVD), and other end processes like ischemic stroke [3]. Multiple epidemiological studies confirm the robust and dose-dependent connection between raised Lp(a) levels and an increased risk of developing cardiovascular events, such as myocardial infarction and stroke, emphasizing the importance of Lp(a) screening as a vital part of overall heart health check-ups [10].

Moreover, there is overwhelming evidence indicating that Lp(a) plays a critical role in the pathogenesis of CVD and that an individual's level of Lp(a) is determined primarily by genetics, with only a slight dependence on lifestyle factors or conventional lipid-lowering therapies [11]. This inherited genetic predisposition leads to a broad spectrum of Lp(a) levels among the population: some individuals may have high Lp(a) levels (exceeding 100 mg/dL) throughout their lifetime (a potential longevity risk factor), while other individuals may exhibit consistently low levels (below 10 mg/dL). Identifying exactly how Lp(a) causes CVD is pivotal to creating targeted therapeutic strategies. Additionally, integrating Lp(a) measurements plus cardiovascular risk assessment algorithms into clinical practice guidelines is necessary to enhance risk prediction accuracy and identify individuals who require more preventive measures or appropriate therapies. 

The inclusion criteria for this review included literature that evaluated the relation between Lp(a) and atherosclerosis and aortic stenosis. A search of PubMed and Google Scholar yielded several articles related to this topic. A literature review of these studies, evaluating chemical properties and structure and, more importantly, the importance of Lp(a) in CVD was conducted (Table 1).

**Table 1:** Results of literature review conducted Lp(a): Lipoprotein(a), LDL: low-density lipoprotein

Authorship & Year of Publication	Objective	Conclusion
Tsimikas et al., 2020, [1]	Report on a clinical trial evaluating Lp(a) reduction in individuals with cardiovascular disease.	The trial demonstrated that an investigational antisense therapy can effectively reduce Lp(a) levels in individuals with established cardiovascular disease.
Koschinsky and Boffa, 2022, [2]	Examine oxidized phospholipid lipoprotein modification's epidemiology, biochemistry, and pathophysiology (a).	Oxidized phospholipids on Lp(a) are pro-inflammatory and may contribute to its atherogenicity and prothrombotic effects
He et al., 2018, [3]	Review lipoprotein lipase and its role in diseases like atherosclerosis.	Lipoprotein lipase plays a crucial role in lipid metabolism, and its dysregulation contributes to developing atherosclerosis and other diseases.
Ferretti et al., 2017, [4]	Explore the role of Lp(a) in atherothrombosis.	Lp(a) likely contributes to atherothrombosis through various mechanisms, including inflammation, oxidation, and coagulation.
Boffa et. al., 2021, [5]	Provide an overarching view of lipoprotein(a).	Lp(a) is a unique lipoprotein with structural similarities to plasminogen, and elevated levels are a significant risk factor for cardiovascular disease.
Khatana et al., 2020, [6]	Provide insights into oxidized LDL-induced atherosclerosis mechanisms.	Oxidized LDL initiates and propagates atherosclerosis through various mechanisms, including endothelial dysfunction, inflammation, and foam cell formation.
Bourgeois et al., 2021, [7]	Using proteomics and transcriptomics to identify biological pathways linking lp(a) to aortic stenosis	Lp(a) may contribute to aortic stenosis through mechanisms involving inflammation, vascular remodeling, and calcification
Boffa et al., 2016, [8]	To determine if Lp(a) is a direct prothrombotic factor in cardiovascular disease.	Lp(a) appears to be a direct prothrombotic risk factor, but its mechanisms in cardiovascular disease require further study.
Tsimikas, 2019, [9]	Explore the potential causality and emerging therapies for Lp(a) and associated oxidized phospholipids in calcific aortic valve stenosis.	Lp(a) and its oxidized phospholipids may contribute to calcific aortic valve stenosis, and emerging therapies targeting these pathways show promise.
Lu et al., 2022, [10]	Explore novel directions for diagnosing and targeting lipoproteins in atherosclerosis.	Lipoproteins play crucial roles in atherosclerosis, and targeting them could lead to improved diagnostic and therapeutic approaches.
Cesaro et al., 2020, [11]	Discuss Lp(a) as a marker for cardiovascular disease and target for therapies.	Lp(a) is a promising therapeutic target and emerging therapies aim to reduce its levels and associated cardiovascular risk
Pantazi et al., 2022, [12]	Provide an update on oxidized phospholipids and lipoprotein-associated phospholipase A2 in atherosclerotic cardiovascular disease.	Oxidized phospholipids and lipoprotein-associated phospholipase A2 are promising biomarkers and potential therapeutic targets in atherosclerotic cardiovascular disease.
Wang et al., 2024, [13]	Explore the role of neutrophil extracellular traps (NETs) as a catalyst for atherosclerosis.	NETs likely contribute to atherosclerosis through various mechanisms, including endothelial dysfunction, inflammation, and thrombosis.
Youssef et al., 202, [14]	Discuss the expanding knowledge on Lp(a) in aortic valve narrowing.	Lp(a) is emerging as an essential risk factor for aortic valve narrowing, and further research is needed to understand its mechanisms and develop targeted therapies.
Rogers and Aikawa, 2015, [15]	Discuss the role of Lp(a) in calcific aortic valve disease development.	Lp(a) likely contributes to calcific aortic valve disease development through various mechanisms, including inflammation and oxidation.
Ugovšek and Šebeštjen, 2021, [16]	Examine Lp(a) at the crossroads of atherosclerosis, atherothrombosis, and inflammation.	Lp(a) plays a role in atherosclerosis, atherothrombosis, and inflammation, and its measurement could aid in cardiovascular risk assessment.
Vinci et al., 2023, [17]	Discuss Lp(a) as a risk factor for cardiovascular diseases, including pathophysiology and treatment perspectives.	Lp(a) is a significant risk factor for cardiovascular diseases, and further research is needed to develop effective therapies targeting lipoprotein(a).
Wang et al., 2023, [18]	Conduct a bibliometric and visualization analysis on Lp(a) in atherosclerosis.	The analysis reveals the increasing research trends and highlights the importance of Lp(a) in atherosclerosis.
Lampsas et al., 2023, [19]	Discuss Lp(a) in atherosclerotic diseases from pathophysiology to diagnosis and treatment.	Lp(a) is a crucial risk factor for atherosclerotic diseases, and its measurement and potential therapies are active research areas
Šuran et al. 2022, [20]	Conduct a bibliometric study on Lp(a) in cardiovascular diseases.	The study highlights the increasing research interest in Lp(a) and its importance in cardiovascular diseases.

Discussion 

Lipoprotein(a) formed the center of a recent cardiovascular study which looked specifically at Lp(a)’s role in the development of atherosclerosis and aortic valve stenosis. Over the last decade, research has shown that raised Lp(a) levels are strongly associated with developing and advancing these diseases through different mechanisms such as inflammation, oxidative stress, and the calcium deposit development processes.

Pathophysiology 

Lp(a) is a critical player in atherosclerosis pathogenesis, exhibiting its impact through multiple mechanisms [3]. Lp(a) is associated with arterial inflammation and oxidative stress, both of which significantly contribute to the growth of atherosclerotic plaques and subsequent arterial narrowing [6]. Research indicates that Lp(a) plays a role in the formation, growth, and progression of unstable atherosclerotic plaques, which are prone to rupture and increase the risk of thrombotic events [2]. Apo(a) possesses a strong lysine binding site essential for the incorporation of oxidized phospholipids (OxPL). OxPL attached to apo(a) contributes to the adverse effects of Lp(a) on various cell types related to atherosclerosis and valve disease, both in vitro and in vivo [2]. Some studies have shown that Lp(a) can effectively trigger the production of proinflammatory cytokines and reactive oxygen species, thereby maintaining an inflammatory state within the arterial wall [12]. Additionally, recent research updates have highlighted the roles of OxPL and lipoprotein-associated phospholipase A2 (Lp-PLA2) in atherosclerotic cardiovascular disease [12]. 

The involvement of Lp(a) in endothelial dysfunction is a critical early step in atherosclerosis development. Recent research has shown that elevated Lp(a) levels can negatively impact endothelial function by interfering with lipoprotein transport, increasing adhesion reactions, and reducing vasodilator function. These changes facilitate the transport and retention of Lp(a) and other atherogenic particles within the intimal layer of the vessels, promoting acute inflammation. Furthermore, studies using a multi-omics approach, incorporating proteomics and transcriptomics, have explored the biological processes through which elevated Lp(a) predicts aortic valve stenosis. Proteomics analysis identified potential proteins linked to Lp(a) particles related to aortic valve stenosis development, including enzymes involved in wound healing, leukocyte migration, platelet degranulation, and protein activation cascades [7]. In calcified aortic valve studies, high Lp(a) levels were found to impact genes controlling inflammation, chondrocyte development, and cell aging. Although these studies did not find that increased Lp(a) levels directly cause endothelial dysfunction or atherosclerosis progression, they enriched the understanding of how Lp(a) contributes to cardiovascular disease development [6,7]. However, recent research has shown the opposite, indicating that elevated Lp(a) levels can negatively impact endothelial function by interfering with lipoprotein transport, increasing adhesion reactions, and reducing vasodilator function [6]. Such modifications encourage Lp(a) and other atherogenic particles to be transported to and remain within the intimal layer of the vessels, which in turn fuels the acute inflammatory process. In summary, Lp(a) is found to cause atherosclerosis through its role in arterial inflammation, oxidative stress, and endothelial dysfunction, with recent studies highlighting its complex interactions and contributions to cardiovascular disease. 

Furthermore, research has shown the presence of Lp(a) in plasma foam cells and atherosclerotic plaques, highlighting its role in plaque formation and inflammation [16]. High levels of Lp(a) can damage the endothelial lining by competing with other lipoproteins. Lp(a) anchors and activates an adhesion sequence but fails to promote vasodilation. Additionally, Lp(a) has been found in plasma foam macrophages and atherogenic plaques, underscoring its involvement in inflammation and plaque development [10]. The permeable nature of various cell types allows Lp(a) to be detectable in plasma. Studies have reviewed the role of Lp(a) in atherosclerosis, inflammation, and thrombosis, suggesting that high Lp(a) levels may impair endothelium-dependent cell signaling, facilitate the translocation of atherogenic particles into the intima of blood vessels, and promote inflammation [16].

Lp(a) particles are sufficiently small to penetrate the arterial wall [1]. Research has demonstrated the presence of Lp(a) in plasma foam cells and atherosclerotic plaques, highlighting its significant role in plaque formation and inflammation. Elevated levels of Lp(a) can damage the endothelial lining by competing with other lipoproteins, anchoring and activating adhesion sequences without promoting vasodilation. Additionally, Lp(a) has been identified in plasma foam macrophages and atherogenic plaques, underscoring its involvement in inflammation and plaque development. The permeable nature of various cell types allows Lp(a) to be detectable in plasma. Studies have reviewed the role of Lp(a) in atherosclerosis, inflammation, and thrombosis, suggesting that high Lp(a) levels may impair endothelium-dependent cell signaling, facilitate the translocation of atherogenic particles into the intima of blood vessels, and promote inflammation. Once inside the arterial wall, Lp(a) particles undergo oxidation and interact with macrophages. These modified particles are then absorbed by cells, forming foam cells characteristic of early atherosclerosis. Additionally, Lp(a) recruits and activates inflammatory cells, further exacerbating plaque development. Clinical and population-based studies, including meta-analyses, have shown a positive correlation between Lp(a) levels and the risk of ASCVD. Elevated Lp(a) levels are associated with conditions such as coronary heart disease, myocardial infarction, and ischemic stroke. Lp(a) is statistically linked to ischemic heart disease, aortic stenosis, thromboembolism, and stroke, even when traditional risk factors are considered. Individuals with high Lp(a) levels face a significantly increased risk - two to four times higher - of early-onset ischemic heart and blood vessel diseases compared to those without elevated Lp(a) levels. 

However, it has been shown in clinical and large population-based studies and meta-analyses that Lp(a) has a positive correlation with ASCVD risk [10]. Studies have also discussed elevated levels of Lp(a) that are associated with the conditions that cause coronary heart disease, myocardial infarction, and ischemic stroke [4,11]. Lp(a) is statistically related to ischemic heart disease, aortic stenosis (AS), thromboembolism, and stroke when analyzed with traditional risk factors with or without adjustment. Persons with high Lp(a) levels experience significantly increased chances of early onset ischemic heart and blood vessel diseases 2-4-fold than those who do not have this genetic disease. Ferretti's studies suggest that individuals with elevated Lp(a) concentrations are more likely to develop coronary heart disease, myocardial infarction, or ischemic stroke than those without this risk factor, even after adjusting for other traditional risk factors [11]. Lp(a) is a proven antigenic factor for ASCVD, proved by showing that high levels are causally linked to a higher risk of " ischemic stroke, aortic valve stenosis, and coronary artery disease [11]. Lp(a) engages in the thrombotic and lipid pooling processes responsible for ASCVD. Some studies pointed out the function of Lp(a) in the process of thrombosis, specifically highlighting its ability to interact with plasminogen, making plasminogen inhibit fibrinolysis [5]. Lp(a) is an essential pathogenic factor in AS, a valvular heart disease wherein the aortic valve orifice is narrowed and causes the heart to work harder, requiring more pressure for efficient blood flow [13,14]. These underlying mechanisms are multifaceted and made possible by calcifying aortic valve leaflets and a proinflammatory and oxidative environment within the valve. Lp(a) also participates in the aortic valve leaflet calcification, although the primary mechanism of how Lp(a) leads to aortic valve stenosis still needs to be clarified [13,14].

Lp(a) is also linked to the calcium deposition in the aortic valve leaflets [14]. Research has identified a pathway that links Lp(a), autotaxin, and inflammation to aortic valve calcification [13,14]. Higher levels of Lp(a), autotaxin, oxidized lipids, inflammation, and calcification have been observed in diseased human aortic valves compared to normal valves. In vitro experiments demonstrated that Lp(a) and its oxidized phospholipids induce pro-osteogenic changes in valve interstitial cells through an inflammatory pathway involving nuclear factor kappa-light-chain-enhancer of activated B cells (NF-kB) and bone morphogenetic protein 2 (BMP2) [14]. inhibition of autotaxin or blocking its Lp(a) receptor could be promising therapeutic strategies for aortic valve disease, warranting further investigation. This process results in the thickening and stiffening of the valve leaflets, potentially leading to aortic stenosis. Elevated Lp(a) levels can trigger inflammatory reactions in the aortic valve and may also enhance the oxidation of valve tissue [15]. Likewise, there is also evidence that elevated Lp(a) levels are an autonomous, underlying risk factor for aortic stenosis and calcified aortic valve disease (CAVD) [14]. Genetic studies have identified Lp(a)-raising variants that increase the risk of clinical stenosis and aortic valve calcification by over 50%. Lp(a) may exacerbate CAVD by promoting inflammation, foam cell formation, and endothelial dysfunction, with its oxidized phospholipid content facilitating pro-osteogenic signaling in valve cells. With no current medical therapy for aortic stenosis, the authors suggest lowering Lp(a) using emerging therapies could be the first treatment to halt CAVD progression [14].

Hence, these inflammatory reactions and oxidative stresses play an antagonistic role by damaging the tissues of the valve in more advanced stages of the condition, resulting in severe calcification [16]. Significant scientific findings have elucidated Lp(a)’s role in the pathogenesis of aortic stenosis [9]. Enhanced Lp(a) levels are believed to be responsible for quicker aortic progression to stenosis, as reported by Tsimikas et al. and Vinci et al., whose studies established clinically measurable parameters such as stenotic valve area and trans-valvular gradients [17]. Studies have also proven that Lp(a) plays an essential role in the onset of CAVD, which is one of the first steps in aortic stenosis formation [9,18]. Increased knowledge about the role that Lp(a) plays in CVD development is leading to the development of new therapeutic dimensions for treating CVD [9,15]. Existing methods for treatment include lipid-lowering therapies, which have demonstrated an ability to lessen Lp(a) levels by only a modest degree.

Pharmacological Management 

Studies have examined how statins, fibrates, and many other traditional cholesterol-reducing drugs influence the circulating levels of Lp(a) [20]. The study also highlighted that increased Lp(a) concentrations are independent endogenous risk factors for ASCVD attributable to genetic factors for which LPA gene encoding apo(a) is accountable. However, other studies report that fibrates are a class of drugs that are employed in clinical settings to boost the activity of lipoprotein lipase (LPL) by suppressing the expression of apolipoprotein C-III (APOC3) [3]. Findings show that, although these medications may provide a slight decrease in Lp(a) levels, the results are often insufficiently compelling. This demonstrates a need for medications that can more substantially lower levels of Lp(a) and directly address the problem posed by elevated Lp(a) [4]. New, selective treatments developed specifically for lowering Lp(a) plasma levels promise to be more effective. Tsimikas et al. and Vinci et al. have focused on using antisense oligonucleotides and RNA interference (RNAi) therapies that specifically decrease Lp(a) production in the liver. Enhanced Lp(a) levels are believed to be responsible for quicker aortic progression to stenosis, in studies that establish clinically measurable parameters such as stenotic valve area and trans-valvular gradients. Increased knowledge about the role that Lp(a) plays in CVD development leads to the development of new therapeutic dimensions for treatment [1]. These novel approaches have demonstrated a noteworthy decrease in Lp(a) concentrations, a prerequisite for the successful management of atherosclerosis associated with elevated Lp(a) [9]. On the other hand, Lp(a) levels are often associated with platelet function. These platelet-influencing modifications may lead to alterations in hemostatic regulation and function, including changes in Lp(a) and Apo(a). The hemostatic and platelet-modulating effects of these modifications remain to be evaluated [9]. Possible consequences of developing treatments to successfully lower Lp(a) levels expand beyond aortic stenosis and apply to the broader areas of cardiovascular risk prevention and management. Research specifically addresses Lp(a) in relation to CVDs, especially aortic valve stenosis [17]. However, elevated Lp(a) is directly related to clinical parameters such as stenotic valve area and transvalvular gradients, which are involved in the progression of aortic stenosis. The research showed that Lp(a) is involved with the development of CAVD, which is an initial stage in the development of aortic stenosis. Greater awareness of the role of Lp(a) in cardiovascular diseases means that new strategies for treating conditions that cause high Lp(a) are being sought [17]. Because elevated Lp(a) levels are largely genetic and have been linked to the process of atherosclerosis formation, coronary artery disease, ischemic stroke, and higher rates of heart failure, therapies designed to lower Lp(a) levels could have important implications in preventative treatment plans. Additionally, the ability to effectively lower Lp(a) levels may bring about important advances in personalized medicine and risk stratification [1,9].

## Conclusions

Lipoprotein(a) plays a crucial role in the pathogenesis of cardiovascular disease, particularly atherosclerosis and aortic stenosis. This is due primarily to Lp(a)’s role in contributing to inflammation, oxidative stress, and calcification. The positive correlation between high Lp(a) levels and increased cardiovascular risk highlights the necessity of developing appropriate, effective therapeutic modalities to reduce Lp(a) levels in individual patients. Traditional therapies such as lipid-slacking drugs are largely ineffective at reducing Lp(a) levels. However, modern targeted therapeutics like antisense oligonucleotides and RNA interference have been shown to noticeably reduce Lp(a) levels. As Lp(a) screening and treatments become standard practice, cardiologists will be able to conduct better risk estimates for individual patients and offer more effective, personalized treatments, advances, which will, in turn, lower the overall incidence of Lp(a)-related cardiovascular disease.

## References

[1] Tsimikas S, Karwatowska-Prokopczuk E, Gouni-Berthold I (2020). Lipoprotein(a) reduction in persons with cardiovascular disease. N Engl J Med.

[2] Koschinsky ML, Boffa MB (2022). Oxidized phospholipid modification of lipoprotein(a): epidemiology, biochemistry and pathophysiology. Atherosclerosis.

[3] He PP, Jiang T, OuYang XP (2018). Lipoprotein lipase: biosynthesis, regulatory factors, and its role in atherosclerosis and other diseases. Clin Chim Acta.

[4] Ferretti G, Bacchetti T, Johnston TP, Banach M, Pirro M, Sahebkar A (2018). Lipoprotein(a): a missing culprit in the management of athero-thrombosis?. J Cell Physiol.

[5] Boffa MB, Koschinsky ML (2021). Lipoprotein(a).

[6] Khatana C, Saini NK, Chakrabarti S, Saini V, Sharma A, Saini RV, Saini AK (2020). Mechanistic insights into the oxidized low-density lipoprotein-induced atherosclerosis. Oxid Med Cell Longev.

[7] Bourgeois R, Bourgault J, Despres AA (2021). Lipoprotein proteomics and aortic valve transcriptomics identify biological pathways linking lipoprotein(A) levels to aortic stenosis. Metabolites.

[8] Boffa MB, Koschinsky ML (2016). Lipoprotein (a): truly a direct prothrombotic factor in cardiovascular disease?. J Lipid Res.

[9] Tsimikas S (2019). Potential causality and emerging medical therapies for lipoprotein(A) and its associated oxidized phospholipids in calcific aortic valve stenosis. Circ Res.

[10] Lu Y, Cui X, Zhang L (2022). The functional role of lipoproteins in atherosclerosis: novel directions for diagnosis and targeting therapy. Aging Dis.

[11] Cesaro A, Schiavo A, Moscarella E (2021). Lipoprotein(a): a genetic marker for cardiovascular disease and target for emerging therapies. J Cardiovasc Med (Hagerstown).

[12] Pantazi D, Tellis C, Tselepis AD (2022). Oxidized phospholipids and lipoprotein-associated phospholipase A(2) (Lp-PLA(2)) in atherosclerotic cardiovascular disease: an update. Biofactors.

[13] Wang Y, Wang C, Li J (2024). Neutrophil extracellular traps: a catalyst for atherosclerosis. Mol Cell Biochem.

[14] Youssef A, Clark JR, Koschinsky ML, Boffa MB (2021). Lipoprotein(a): Expanding our knowledge of aortic valve narrowing. Trends Cardiovasc Med.

[15] Rogers MA, Aikawa E (2015). A not-so-little role for lipoprotein(a) in the development of calcific aortic valve disease. Circulation.

[16] Ugovšek S, Šebeštjen M (2021). Lipoprotein(a)-the crossroads of atherosclerosis, atherothrombosis and inflammation. Biomolecules.

[17] Vinci P, Di Girolamo FG, Panizon E (2023). Lipoprotein(a) as a risk factor for cardiovascular diseases: pathophysiology and treatment perspectives. Int J Environ Res Public Health.

[18] Wang H, Pan D, Liao L (2023). Lipoprotein (a) in atherosclerosis: a bibliometric and visualization analysis. Vasc Investig Ther.

[19] Lampsas S, Xenou M, Oikonomou E (2023). Lipoprotein(a) in atherosclerotic diseases: from pathophysiology to diagnosis and treatment. Molecules.

[20] Šuran D, Blažun Vošner H, Završnik J (2022). Lipoprotein(A) in cardiovascular diseases: insight from a bibliometric study. Front Public Health.

